# Self-inflicted neck wounds under influence of lysergic acid diethylamide: A case report and literature review

**DOI:** 10.1097/MD.0000000000020868

**Published:** 2020-07-02

**Authors:** Brendan Le Daré, Thomas Gicquel, Alain Baert, Isabelle Morel, Renaud Bouvet

**Affiliations:** a Institut national de la santé et de la recherche médicale, Institut national de recherche agronomique, University Hospital Rennes, Institut NuMeCan (Nutrition, Metabolism and Cancer); b Forensic Toxicology Laboratory; c Department of Forensic Medicine, Rennes University Hospital; d Institut du droit public et de la science politique - EA 4640, University Rennes, Rennes, France.

**Keywords:** case report, lysergic acid diethylamide, neck wounds, self-inflicted trauma

## Abstract

**Rationale:**

Lysergic acid diethylamide (LSD) is a highly potent psychedelic drug derived from ergot alkaloids. The available literature data derived from controlled studies or usage in a medical setting seem reassuring; however the literature contains very rare cases of fatal self-inflicted injuries associated with LSD exposure. The behavioral disorder that created the conditions conducive to death is a maladaptive or irrational response to the psychiatric manifestations induced by the substance.

**Patient concern:**

Here, we report the case of a 26-year-old man found dead with large neck wounds in a locked house. No medical history other than recreational use of alcohol and narcotics was reported as well as any history of psychotic disease. The entirety of the other investigations carried out did not demonstrate the presence of a third party at the place of death and a dropper bottle containing LSD was found near the body.

**Diagnosis:**

We report the first case of fatal self-inflicted neck wounds with a cutting instrument in the context of acute exposure to LSD in a patient with no psychiatric history and without suicidal symptoms at the time of the self-aggressive act.

**Intervention and outcomes:**

In the present work, we used a validated method using liquid chromatography coupled with mass spectrometry for simultaneous quantification of LSD and its metabolites (O-H-LSD and Nor-LSD) in whole blood and urine samples. LSD and O-H-LSD were respectively found at 1460 and 182 pg/mL in blood. In the urine, the concentrations of LSD, nor-LSD, O-H-LSD were, respectively, 3670, 201, and 4890 ng/L.

**Lessons:**

This observation is particularly relevant in view of the resurgence of interest in the therapeutic use of LSD, notwithstanding the fact that the literature has not demonstrated a link between suicidal risk and acute or chronic exposure to LSD.

## 1 Introduction

Lysergic acid diethylamide (LSD) is a well-known psychoactive substance that is once again the subject of intense research efforts, after previous efforts in the 1970s were abandoned when it was classified as a narcotic.^[1–4]^ The safety of the molecule warrants evaluation in view of its use as an adjunct to psychotherapy. The available literature data derived from controlled studies or usage in a medical setting seem reassuring; however the medicolegal literature contains rare cases of accidental death associated with LSD exposure and very rare cases of self-inflicted injuries that were fatal. We report the first case of a lacerating penetrating neck injury and discuss the self-inflicted nature of the injury and also the role of acute exposure to LSD in the self-aggressive act.

## 2 Case presentation

**Figure 1:**
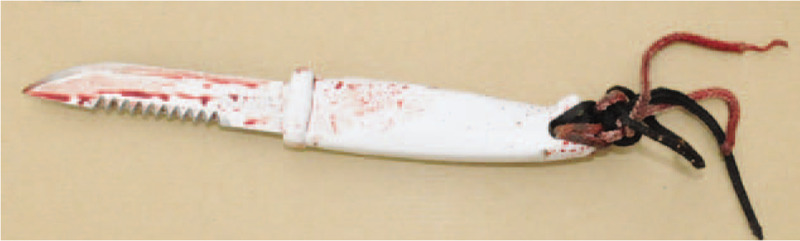
Knife found in the immediate vicinity of the body.

**Figure 2:**
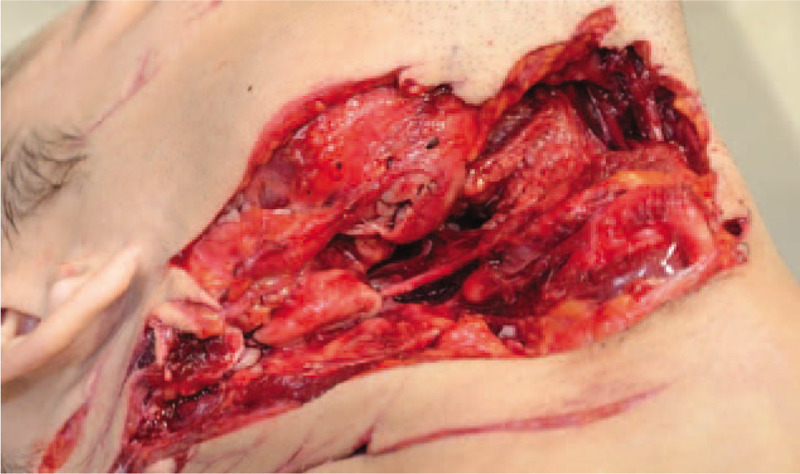
Large neck wounds presenting muscle wounds, wounds to the oropharynx, and incomplete section of the right transverse process of the first cervical vertebra.

The body of a 26-year-old man with large neck wounds was discovered at his grandfather's locked house at 8.30 am. The criminal investigation technicians noted the presence of blood on the floor and on the walls, and a knife with a blade 10 cm long and 1.5 cm wide, double-edged with one smooth edge and one serrated edge, and a sharp tip (Fig. 1). The time of death as estimated by the forensic medical examiner was 4 am on the same day. The autopsy performed 7 hours after the discovery of the body found 1 wound to the face and 5 straight neck wounds inflicted by a cutting instrument, with wounds being linked. Two of the neck wounds were particularly lacerated (Fig. 2); one of them was fatal via sectioning of the right internal carotid artery and right common facial vein. Exploration of the neck also showed, in addition to muscle wounds, wounds to the oropharynx and incomplete section of the right transverse process of the first cervical vertebra.

Full toxicological analyses were performed. Ethanol was found at a concentration of 0.88 g/L in blood. The LSD and its metabolites (Nor-LSD and O-H-LSD) were quantified using a specific and sensitive LC-MS/MS method. In blood, LSD and O-H-LSD were found at concentrations of 1460 and 182 ng/L respectively. In urine, the concentrations of LSD, nor-LSD, and O-H-LSD were 3670, 201, and 4890 ng/L respectively.

There is no reported medical history other than recreational use of alcohol and narcotics. There is no history of psychotic disease and his behavior was described as normal up to 11 pm by his father, with whom he had exchanged some text messages. The inquiry revealed that on his request, his father had provided to him the day before a dropper bottle containing a liquid, which was shown by the toxicology analysis to be LSD at a concentration of 2 mg/mL. The entirety of the other investigations carried out (including telephone audio recordings) did not demonstrate the presence of a third party at the place of death. The responsibility of the grandfather, the only other person present at the location, was ruled out. Taken together, these elements led to the conclusion that the injuries were self-inflicted in the context of acute exposure to LSD.

## 3 Discussion and conclusion

We report here the first case of a fatal self-inflicted neck injury with a cutting instrument in the context of acute exposure to LSD in a patient with no known psychiatric history or suicidal symptoms at the time of the self-aggressive act. Given the resurgence of interest in the therapeutic use of LSD, this observation should be taken into account, even if the literature has not provided evidence of a link between suicidal risk and acute or chronic LSD exposure.

LSD was synthesized for the first time in 1938. Initially, there were no obvious therapeutic applications. After Hofmann accidentally discovered its psychoactive properties,^[5]^ recreational use became quite widespread in the 1960s, and it was used for medical purposes in psychiatry, until it was classified as a narcotic in 1971 owing to its adverse events.^[6]^ Like other hallucinogens, the frequency of experimentation with LSD remains stable at a low level (1.7% of people aged 18–64 years in France between 2000 and 2010, vs 3.1% for hallucinogenic mushrooms),^[7]^ although there are significant differences between countries (10.2% in the United States of America).^[4]^ In parallel with illicit use, current medical research also seems to be turning its attention to LSD once again,^[3]^ notably as an adjunct to psychotherapy.^[1,2]^ This accounts for recent work looking at its pharmacology,^[8,9]^ techniques for quantifying the molecule and its metabolites,^[10]^ and its safety.^[3,11]^ The resurgence of interest in the molecule provides the impetus for study into the adverse events associated with acute or chronic exposure to LSD, and here, medicolegal analysis of deaths provides information beyond that obtained during controlled administration of the substance in research settings.^[12]^ The medicolegal contribution to the study of the epidemiology of adverse events following LSD exposure is essentially the analysis of the causes of and circumstances surrounding death. Two situations present themselves: “direct” LSD toxicity (intoxication causing death) and “indirect” toxicity, in which the substance creates the conditions conducive to death, notably behavioral disorders (intoxication as a circumstance of death).

In relation to direct toxicity, Nichols and Grob^[13]^ consider that the published cases of death are insufficient to establish a link between LSD exposure and death. Apart from the issue of the pertinence of postmortem blood concentrations in determining cause of death, which today seems highly questionable,^[14]^ analysis of these cases shows that the deaths reported in the literature occurred in subjects questioned by the police and placed in the prone maximum restraint (PMR) position. Given that the circumstances could have played a crucial role in causing death, the authors consequently concluded that there are no reported cases of bad LSD trips leading to fatality, instead putting forward the hypothesis that PMR can lead to fatality in people having a bad LSD trip, which is a significant nuance.

In relation to indirect toxicity linked to LSD-induced behavioral disorders, 2 situations can be envisaged. The first is impairment of the subject's perception of reality, with death not being directly related to the substance. This was the mechanism in 5 cases of people jumping from a height (one of which followed exposure to lysergamide, a non-alkylated amide-analog of LSD),^[15–17]^ and 2 cases of drowning.^[18]^ The jumps were attributed to a sensation of weightlessness giving the subject the impression of being able to fly.

The second situation concerns self-inflicted injuries, which are possibly fatal. The issue is then whether LSD exposure leads to such behavior. Liechti^[3]^ observed that in healthy subjects, the LSD experience was not dominated by unpleasant psychosis-like phenomena but rather characterized by an overall positive mood state in the majority of subjects. Subjects were described as more distant from reality and happy after administration of 200 μg LSD, whereas ratings of anxiety and paranoid thinking did not increase. In other words, acute exposure does not seem to bring about symptoms conducive to aggressive acts, including self-aggressive acts. With regard to chronic exposure, Hendricks et al^[4]^ showed, on the basis of a study in 190,000 adult American subjects, that lifetime classic psychedelic use was associated with significantly reduced odds of past-month psychological distress, past-year suicidal thinking, past-year suicidal planning, and past-year suicide attempt.

The literature contains 4 cases of severe self-inflicted injuries associated with LSD exposure: 3 enucleations and 1 emasculation. Yet the role of LSD in these 4 cases warrants discussion. In the case of enucleation reported by Tuwir,^[19]^ the blood and urine toxicology analysis demonstrated the presence of amphetamine, but presence of LSD was not sought; therefore this case is insufficiently documented in toxicology terms. The 2 cases of enucleation reported by Rosen and Hoffman^[20]^ concern 2 young psychotic patients who developed a mystical delusion and intense feelings of guilt after sexual intercourse, with mutilation presented as a redemptive sacrifice. In these cases, the contribution of mental illness to the self-aggressive act is substantial, and one cannot attribute the act exclusively to LSD exposure. Only the case reported by Blacha et al^[21]^ appears to be of real interest, in that it concerns manual amputation of both testes after first use of LSD in combination with alcohol consumption in a 32-year-old man with no psychiatric history and whose follow-up examination 6 months after the event did not reveal the development of a psychiatric disorder.

Hence, cases of severe self-inflicted injuries associated with documented LSD exposure are very rare after psychotic disorders are excluded. Recent works seem to show that acute or chronic LSD exposure does not carry a risk of a self-aggressive act. In our case, the cause of death is, without a shadow of a doubt, the penetrating neck injury that damaged blood vessels. Therefore, LSD exposure cannot be put forward as directly responsible for the death. Rather the issue is whether it created conditions conducive to the self-aggressive act.

To answer this question, first and foremost it is necessary to establish the self-inflicted nature of the injuries. Fatal penetrating neck injuries are relatively rare, and generally result from vascular injury.^[22]^ Cases of suicide by neck wounding are also rare, but regularly described in the medicolegal literature.^[23–26]^ Several publications have addressed the issue of determining the suicidal or homicidal nature of injuries caused by a cutting instrument. Although morphological criteria can lend support to suicide (accessibility, symmetry, etc), they do not provide definitive evidence. Thus, Krywanczyk and Shapiro^[27]^ consider that no characteristics of blade wounds are definitive for homicide or suicide. History and the circumstances of the scene are thus crucial in determining the manner of death. In our case, the neck wounds were significant and deep, which is why a criminal hypothesis was initially favored. Nevertheless, from a strictly medicolegal viewpoint: the damaged area was accessible to a right-handed subject; “hesitation” wounds were observed peripheral to the wounds; no defensive injuries were demonstrated in the upper limbs. In terms of feasibility, the subject could have self-inflicted these injuries up to the moment he sectioned his right internal carotid artery. Moreover, none of the investigations carried out as part of this particularly in-depth inquiry have demonstrated the presence of a third party on the premises. These elements lead us to conclude that the injuries were self-inflicted.

With regard to the mechanism underlying the act, it has been shown that mental illness plays a major role in explaining suicides with a bladed weapon. In the series published by Badger et al,^[28]^ 98% of the subjects met the criteria for a formal psychiatric diagnosis with 89% necessitating inpatient or outpatient psychiatric assistance at discharge. In our case, review of the subject's medical record did not find any evidence in favor of a mental disorder or psychiatric history. The inquiry showed that the subject's mental state in the hours preceding his death was normal, on the basis of the exchanges with his father via text messages. There are therefore no grounds for evoking suicidal symptomatology at the time of the events. The hypothesis of the influence of a psychoactive substance therefore deserves consideration. The influence of ethanol, at this concentration and considering the subject's consumption habits, can be excluded. The blood and urine toxicology analysis only demonstrated the presence of LSD, which was obviously consumed from the dropper bottle discovered on the premises. Given that the complete and efficient toxicology analysis (liquid chromatography tandem-mass spectrometry and liquid chromatography-high resolution mass spectrometry) showed absence of any other psychoactive substances, it seems reasonable to consider the influence of LSD. The above-mentioned literature did not find any association between acute and chronic exposure to LSD and an elevated risk of suicide, a finding which does not contradict our observation. One must distinguish between suicide and a self-aggressive act that may be devoid of suicidal intent and that may constitute a maladaptive or irrational response to the psychiatric manifestations induced by the substance.

## Author contributions

All authors contributed and agree with the content of the manuscript:

**Conception/Design:** Brendan Le Daré, Thomas Gicquel, Renaud Bouvet;

**Acquisition and/or analysis of data:** All authors;

**Data interpretation:** All authors;

**Manuscript writing:** All authors;

**Final approval of manuscript:** All authors.

## References

[1] Gasser P Holstein D Michel Y Safety and efficacy of lysergic acid diethylamide-assisted psychotherapy for anxiety associated with life-threatening diseases. J Nerv Ment Dis 2014;202:513–20. 24594678 10.1097/NMD.0000000000000113 PMC4086777

[2] Gasser P Kirchner K Passie T LSD-assisted psychotherapy for anxiety associated with a life-threatening disease: a qualitative study of acute and sustained subjective effects. J Psychopharmacol 2015;29:57–68. 25389218 10.1177/0269881114555249

[3] Liechti ME Modern clinical research on LSD. Neuropsychopharmacology 2017;42:2114–27. 28447622 10.1038/npp.2017.86 PMC5603820

[4] Hendricks PS Thorne CB Clark CB Classic psychedelic use is associated with reduced psychological distress and suicidality in the United States adult population. J Psychopharmacol 2015;29:280–8. 25586402 10.1177/0269881114565653

[5] Hofmann A LSD: My Problem Child. Trans. Jonathan Ott. New York: McGraw; 1979.

[6] Gicquel T Lepage S Morel I LSD history: From ergot to therapeutic applications. Presse Médicale 2015;44:832–6. 10.1016/j.lpm.2015.04.033 26071028

[7] Beck F Bonnet N The substance or the eventful history of LSD. Médecine/Sciences 2013;29:430–3. 10.1051/medsci/2013294018 23621940

[8] Dolder PC Schmid Y Haschke M Pharmacokinetics and concentration-effect relationship of oral LSD in humans. Int J Neuropsychopharmacol 2016;19:yv072. 10.1093/ijnp/pyv072 PMC4772267 26108222

[9] Dolder PC Schmid Y Steuer AE Pharmacokinetics and pharmacodynamics of lysergic acid diethylamide in healthy subjects. Clin Pharmacokinet 2017;56:1219–30. 28197931 10.1007/s40262-017-0513-9 PMC5591798

[10] Dolder PC Liechti ME Rentsch KM Development and validation of an LC-MS/MS method to quantify lysergic acid diethylamide (LSD), iso-LSD, 2-oxo-3-hydroxy-LSD, and nor-LSD and identify novel metabolites in plasma samples in a controlled clinical trial. J Clin Lab Anal 2018;32:e22265. 10.1002/jcla.22265 PMC6817285 28548305

[11] Schmid Y Enzler F Gasser P Acute effects of lysergic acid diethylamide in healthy subjects. Biol Psychiatry 2015;78:544–53. 25575620 10.1016/j.biopsych.2014.11.015

[12] Drummer OH Kennedy B Bugeja L Interpretation of postmortem forensic toxicology results for injury prevention research. Inj Prev 2013;19:284–9. 23197673 10.1136/injuryprev-2012-040488

[13] Nichols DE Grob CS Is LSD toxic? Forensic Sci Int 2018;284:141–5. 29408722 10.1016/j.forsciint.2018.01.006

[14] Gill JR Stajíc M Classical mistakes in forensic toxicology made by forensic pathologists. Acad Forensic Pathol 2012;2:228–34.

[15] Holding TA Barraclough BM Psychiatric morbidity in a sample of accidents. Br J Psychiatry 1977;130:244–52. 843773 10.1192/bjp.130.3.244

[16] Smith RN Robinson K Body fluid levels of lysergide (LSD). Forensic Sci Int 1985;28:229–37. 4065782 10.1016/0379-0738(85)90133-1

[17] Klinke HB Müller IB Steffenrud S Two cases of lysergamide intoxication by ingestion of seeds from Hawaiian Baby Woodrose. Forensic Sci Int 2010;197:e1–5. 20018470 10.1016/j.forsciint.2009.11.017

[18] Mardal M Johansen SS Thomsen R Advantages of analyzing postmortem brain samples in routine forensic drug screening—case series of three non-natural deaths tested positive for lysergic acid diethylamide (LSD). Forensic Sci Int 2017;278:e14–8. 28803722 10.1016/j.forsciint.2017.07.025

[19] Tuwir I Chako E Brosnahan D Drug induced autoenucleation with resultant chiasmal damage. Br J Ophthalmol 2005;89:121. 15615760 10.1136/bjo.2004.049676 PMC1772460

[20] Rosen DH Hoffman AM Focal suicide: self-enucleation by two young psychotic individuals. Am J Psychiatry 1972;128:1009–12. 4109987 10.1176/ajp.128.8.1009

[21] Blacha C Schmid MM Gahr M Self-inflicted testicular amputation in first lysergic acid diethylamide use. J Addict Med 2013;7:83–4. 23222128 10.1097/ADM.0b013e318279737b

[22] Nason RW Assuras GN Gray PR Penetrating neck injuries: analysis of experience from a Canadian trauma centre. Can J Surg 2001;44:122–6. 11308235 PMC3695107

[23] Misiak P Jabłoński S Dziwińska K A very unusual case of attempted suicide. Kardiochir Torakochirurgia Pol 2016;13:145–7. 27516789 10.5114/kitp.2016.61050 PMC4971271

[24] Ventura F Bonsignore A Gallo M A fatal case of suicidal stabbing and cutting. J Forensic Leg Med 2010;17:120–2. 20211449 10.1016/j.jflm.2009.12.006

[25] Kaliszan M Kernbach-Wighton G BouHaidar R Multiple self-inflicted stab wounds to neck, chest and abdomen as a unique manner of suicide. J Forensic Sci 2010;55:822–5. 20202068 10.1111/j.1556-4029.2010.01322.x

[26] Shetty BSK Padubidri JR Bhandarkar AM “Atypical Suicidal” cut throat injury - a case report. J Forensic Leg Med 2009;16:492–3. 19782325 10.1016/j.jflm.2009.07.003

[27] Krywanczyk A Shapiro S A retrospective study of blade wound characteristics in suicide and homicide. Am J Forensic Med Pathol 2015;36:305–10. 26230455 10.1097/PAF.0000000000000188

[28] Badger JM Gregg SC Adams CA Non-fatal suicide attempt by intentional stab wound: clinical management, psychiatric assessment, and multidisciplinary considerations. J Emerg Trauma Shock 2012;5:228–32. 22988400 10.4103/0974-2700.99688 PMC3440888

